# Supplementary material for: Auxin Regulates Apical Stem Cell Regeneration and Tip Growth in the Marine Red Alga Neopyropia yezoensis
Source: Cells. 2022 Aug 26;11(17):2652. doi: 10.3390/cells11172652 (PMC9454478; doi:10.3390/cells11172652)
Supplement: Supplementary file 1 [file cells-11-02652-s001.zip › cells-1884253-supplementary.pdf]

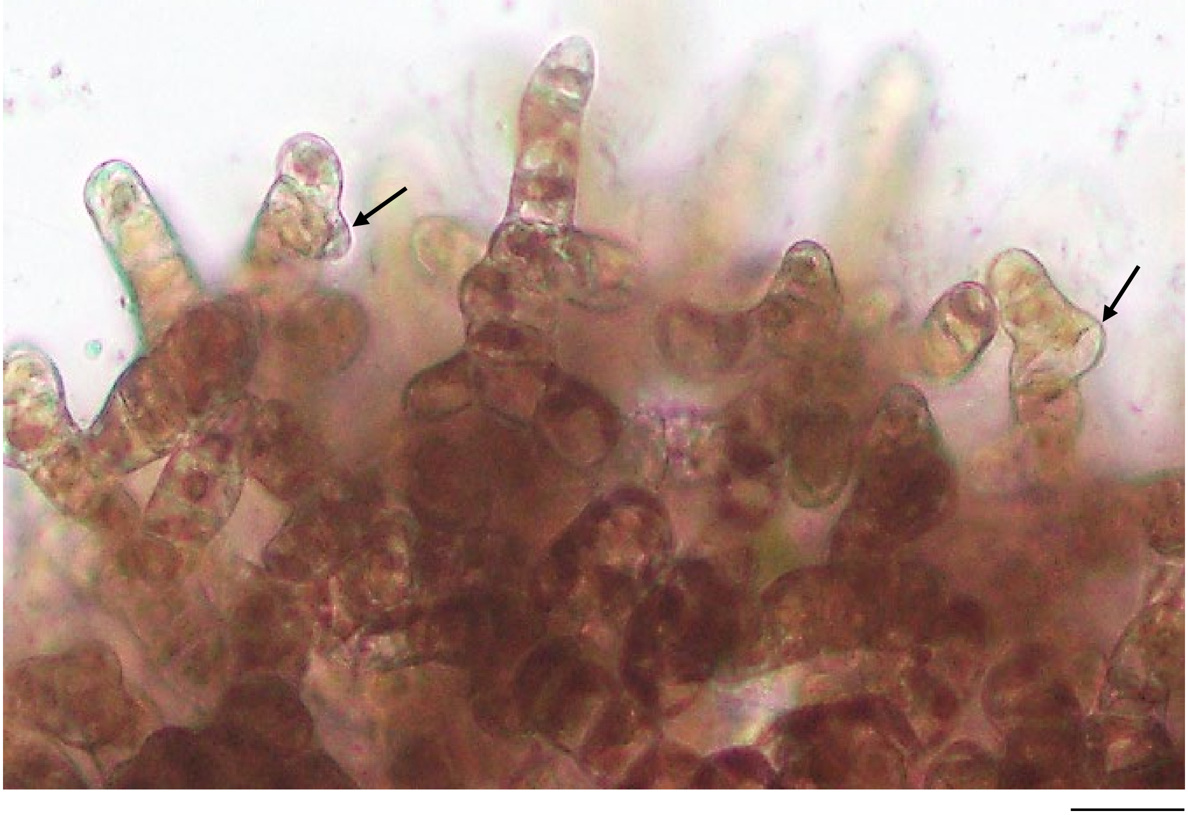

Figure S1. Conchosporangia of *Neopyropia yezopensis*. Clearly visualized side branches are indicated by arrows. Scale bar: 25 μm.

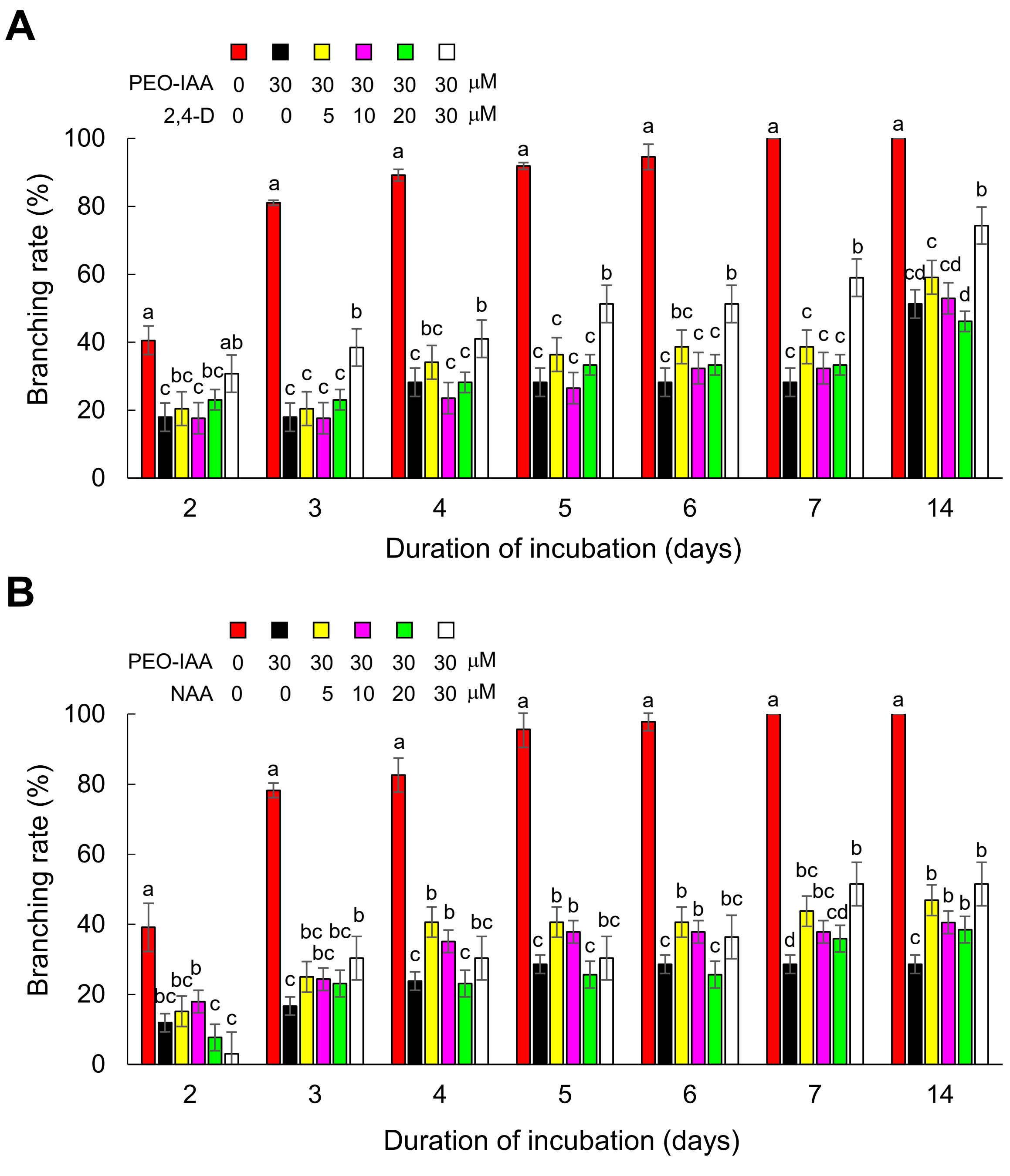

Figure S2. Recovery from inhibitory effects of PEO-IAA by exogenously supplied 2,4-D and NAA. Changes in branching rate in single-celled conchocelis by treatment with various concentration of 2,4-D **(A)** and NAA **(B)** in the presence of 30 μM PEO-IAA are shown. Error bars indicate the standard deviation of triplicate experiments (n = 3), and different lowercase letters denote significant differences in the branching rate, as determined by Tukey's test ($p < 0.05$) for each set of incubation time.

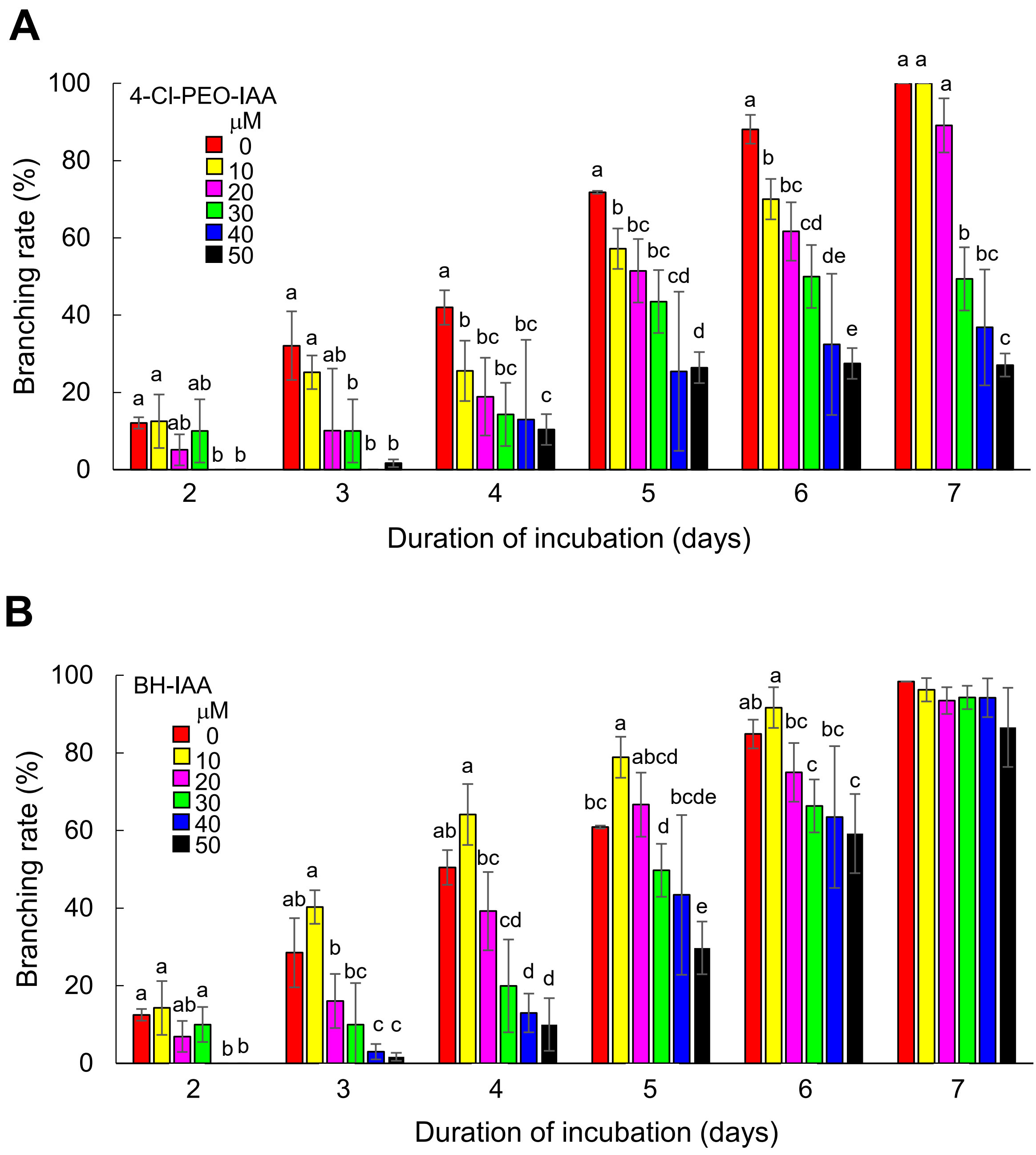

Figure S3. Effects of treatment with the auxin antagonists 4-Cl-PEO-IAA and BH-IAA on the production of the side branches in single-celled conchocelis. Changes in branching rate in single-celled conchocelis following treatment with various concentration of 4-Cl-PEO-IAA **(A)** and BH-IAA **(B)** are shown. Error bars indicate the standard deviation of triplicate experiments (n = 3), and different lowercase letters denote significant differences in branching rate, as determined by Tukey's test ($p < 0.05$) for each set of incubation time.
